# HCoV-NL63 and HCoV-HKU1 seroprevalence and its relationship with the clinical features of COVID-19 patients from Villavicencio, Colombia

**DOI:** 10.7705/biomedica.7168

**Published:** 2024-08-29

**Authors:** Lida Carolina Lesmes-Rodríguez, Luz Natalia Pedraza-Castillo, Dumar Alexander Jaramillo-Hernández

**Affiliations:** 1 Departamento de Biología y Química, Facultad de Ciencias Básicas e Ingeniería, Universidad de los Llanos, Villavicencio, Colombia Universidad de los Llanos Departamento de Biología y Química Facultad de Ciencias Básicas e Ingeniería Universidad de los Llanos Villavicencio Colombia; 2 Escuela de Ciencias Animales, Facultad de Ciencias Agropecuarias y Recursos Naturales, Universidad de los Llanos, Villavicencio, Colombia Universidad de los Llanos Facultad de Ciencias Agropecuarias y Recursos Naturales Universidad de los Llanos Villavicencio Colombia

**Keywords:** SARS-CoV-2, COVID-19, coronavirus NL63, human, betacoronavirus, immunoassay, SARS-CoV-2, COVID-19, coronavirus humano NL63, betacoronavirus, inmunoensayo

## Abstract

**Introduction.:**

Due to the cross-reactivity between SARS-CoV-2 and common human coronaviruses, previous infections with these viruses could contribute to serological or cellular cross-protection against severe COVID-19. However, protective immunity may not develop, or pre-existing immunity could increase COVID-19 severity.

**Objective.:**

To determine the seroprevalence of IgG antibodies against HCoV-NL63 and HCoV-HKU1 and correlate previous exposure with COVID-19 signs in patients from Villavicencio.

**Materials and methods.:**

A cross-sectional retrospective study was conducted. ELISA technique was used to search for IgG antibodies against HCoV-NL3 and HCoV-HKU1 in patients with positive RT-qPCR results for SARS-CoV-2. Patients were grouped according to COVID-19 clinical characteristics in four groups: group 1: asymptomatic (n = 23); group 2: hospitalized (n = 24); group 3: intensive care units (n = 24), and group 4: dead (n = 22).

**Results.:**

The overall seroprevalence of IgG antibodies against HCoV was 74.2% (n = 69; 95% CI: 65.3-83.1), with 66.7% of HCoV-NL63 (n = 62; 95% CI: 57,1-76,2), and 25.8% of HCoV-HKU1 (n = 24; 95% CI: 16,9-34,7). Based on crosstab analysis, prior exposure to HCoV-NL63 was associated with protection against severe COVID-19 (p = 0.042; adjusted OR = 0.159; 95% CI: 0.027-0.938), and previous coinfection of HCoV-NL63 and HCoV-HKU1 was considered a positive association to severe COVID-19 (p = 0.048; adjusted OR = 16.704; 95% CI: 1.020 - 273.670).

**Conclusion.:**

To our knowledge, this is the first study addressing seroprevalence of HCoV IgG antibodies in Colombia and Latin America. Previous exposure to HCoV-NL63 could protect against severe COVID-19, whereas patients with underlying HCoV-NL63 and HCoV-HKU1 coinfection could be hospitalized with severe signs of COVID-19.

## RESUMEN

**Introducción.:**

Debido a la reactividad cruzada entre SARS-CoV-2 y los coronavirus humanos comunes, las infecciones previas con estos virus podrían contribuir a la protección cruzada serológica o celular contra la COVID-19 grave. Sin embargo, la inmunidad protectora puede no desarrollarse o la inmunidad preexistente podría generar COVID-19 grave.

**Objetivo.:**

Determinar la seroprevalencia de anticuerpos IgG frente a HCoV-NL63 y HCoV-HKU1, y correlacionar su previa exposición con los signos de COVID-19 en pacientes de Villavicencio.

**Materiales y métodos.:**

Se realizó un estudio retrospectivo observacional analítico y transversal. Se utilizó la técnica ELISA para buscar anticuerpos IgG contra HCoV-NL3 y HCoV-HKU1 en pacientes con resultado positivo de RT-qPCR para SARS-CoV-2. Los pacientes se agruparon según los signos de COVID-19 en cuatro grupos: grupo 1: asintomáticos (n = 23); grupo 2: hospitalizados (n = 24); grupo 3: unidad de cuidados intensivos (n = 24), y grupo 4: fallecidos (n = 22).

**Resultados.:**

La seroprevalencia general de IgG anti-HCoV fue de 74.2 % (n = 69; IC_95%_: 65,3-83,1), con 66,7 % de HCoV-NL63 (n = 62; IC_95%_: 57,1-76,2) y 25,8 % de HCoV-HKU1 (n = 24; [IC_95%_:16,9-34,7). Según el análisis de las tablas de contingencia, la exposición previa a HCoV-NL63 se asoció con protección de una COVID-19 grave (p = 0,042; OR ajustado = 0,159; IC_95%_: 0,027-0,938) y la previa coinfección de HCoV-NL63 y HCoV-HKU1 se asoció con padecimiento de signos clínicos graves por COVID-19 (p = 0,048; OR ajustado = 16,704; IC_95%_: 1,020- 73,670).

**Conclusión.:**

Según la literatura revisada hasta la fecha, este es el primer estudio sobre la seroprevalencia de anticuerpos IgG de HCoV en Colombia y Latinoamérica. La exposición previa a HCoV-NL63 podría proteger contra la COVID-19 grave, mientras que los pacientes con coinfección subyacente de HCoV-NL63 y HCoV-HKU1 podrían resultar hospitalizados con signos graves de COVID-19.

Common human coronaviruses (HCoV) are RNA viruses belonging to the Coronavirinae ^1^^)^ subfamily. The alphacoronavirus (α-CoV) genus contains NL63 (HCoV-NL63) and 229E (HCoV-229E), whereas the betacoronavirus (ßCoV) genus contains HKU1 (HCoV-HKU1) and OC43 (HCoV-OC43) ^2^. Coronaviruses 229E and OC43 were detected in 1966 and 1967, respectively ^1^. In 2003, an outbreak of SARS-CoV (ßCoV) arose, causing more than 8,000 severe infections with a mortality rate of 10% ^3^. After that outbreak, it was necessary to increase efforts to identify and characterize new coronaviruses after determining they could evolve into complex pathologies. Those efforts resulted in the identification of NL63 ^4^ and HKU1 ^5^ coronaviruses between 2004 and 2005, and of the severe Middle East respiratory syndrome MERS-CoV (ßCoV) in 2012 ^6^. Subsequently, in December 2019, the coronavirus 2 associated with severe acute respiratory syndrome (SARS-CoV-2) (ßCoV) ^7^^)^ emerged. Its rapid transmission -largely due to the high rate of asymptomatic patients and the late detection of signs in symptomatic patients- suddenly caused the coronavirus disease 2019 (COVID-19) pandemic ^8^, causing 6.8 million deaths worldwide and more than 676 million cases ^9^, in addition to incalculable economic losses. Clinical signs caused by the SARS-CoV-2 virus range from mild, such as fever, cough, and myalgia, to severe pneumonia, acute respiratory distress syndrome, and death ^10^.

Acute respiratory infections caused by HCoV represent approximately 15% of common flu in adults, depending mainly on the geographical area, the type of population, and the viral detection techniques ^11^. They are also characterized by a marked seasonality with high rates in the winter months ^11^^,^^12^ and frequently detected in coinfection with other respiratory viruses ^13^, especially affecting young children, older adults, and immunocompromised patients ^5^. Several epidemiological studies addressing acute HCoV infections have been conducted worldwide ^14^^-^^18^. Cross-reactivity has been detected between conserved antigenic domains, including the S2 subunit of the spike protein -the nucleocapsid protein of SARS-CoV-2- and other HCoV ^19^^-^^22^. Therefore, previous infections with HCoV probably contribute to serological or cellular cross-protection against the severity of SARS-CoV-2 infection ^23^. However, while most acute HCoV infections could result in the development of protective immunity, available information also suggests the possibility that adaptive immune responses may fail, and protective immunity may not develop ^19^. This pre-existing immunity could generate severe COVID-19 due to phenomena such as the original antigenic sin ^24^. It is worth mentioning that the production of sub-neutralizing cross-reactive antibodies could exacerbate the inflammatory response, facilitating the entry of SARS-CoV-2 into host cells ^25^.

The goal of the present study was to determine the seroprevalence of IgG antibodies against two common coronaviruses -HCoV-NL63 and HCoV-HKU1- in human serum from patients of Villavicencio (Colombia), with a positive result of quantitative reverse transcription polymerase chain reaction (RT-qPCR) for SARS-CoV-2 and its association with the severity or absence of COVID-19 clinical signs. To the authors' knowledge, this is the first study conducted in Colombia and Latin America addressing the seroprevalence of IgG antibodies against seasonal coronaviruses.

## Materials and methods

### Study design and population

This cross-sectional retrospective study was conducted with a reference population composed of patients previously exposed to SARS-CoV-2. These patients, with positive RT-qPCR results for COVID-19, were part of the official data of the *Secretaría de Salud de Villavicencio* on August 17, 2020, the first wave of COVID-19 in Colombia.

### Sample size

The sample size was estimated with the formula n = [DEFF * *Np* (1 - *p*)] / [(*d* ^2^ / *Z* ^2^_1- α / 2_*(*N* - *1*) + *p* * (1 - *p*)] (Duffau,1999) Esta cita no está incluida en referencias, where: DEFF = design effect, *N* = population size, *p* = anticipated frequency, *d* = confidence limits expressed in percentage, and Z = confidence level according to the standard normal distribution. The software used was OpenEpi (Open-Source Epidemiologic Statistics for Public Health), versión 3.01, provided by the Centers for Disease Control and Prevention (CDC) (http://www.openepi.com/SampleSize/SSPropor.htm). The Z value was 1.96, and the expected probability (p) was established at 1.4% (0.014), according to the epidemiological studies of common coronaviruses in the Americas reported by Kanwar *et al.* ^17^ and Cabeça *et al.* ^18^, who found a prevalence of 1.6% for HCoV-HKU1 in the United States of America, and of 1.2% for HCoV-NL63 and HCoV-OC43 in Brazil, respectively, using a probability of 1.6%. The population of the present study was composed of 3,692 individuals officially diagnosed with positive results for SARS-CoV-2 on August 17, 2020 ^26^ in Villavicencio, Meta, Colombia, excluding data from the city prison. The sample size was 93 patients using a 95% confidence interval (CI).

### Sampling and inclusion criteria

Random two-stage cluster probabilistic sampling was performed for the present study. The random selection was carried out among the COVID-19 patients at the *Hospital Departamental de Villavicencio.* Four clusters were formed considering the absence or the severity of the COVID-19 signs in the patients as follows: group 1: asymptomatic patients (n = 23); group 2: hospitalized patients with moderate signs (n = 24); group 3: patients hospitalized in intensive care units with severe symptoms (n = 24), and group 4: dead patients (n = 22). A simple random sampling was performed in each cluster.

Patients included in the study aged 18 years or older, habitually residing in the city of Villavicencio and diagnosed with an RT-qPCR test according to the official COVID-19 statistics from the databases of the *Secretaría de Salud de Villavicencio.* Patients with negative RT-qPCR results for SARS-CoV-2 and pregnant women were not included in the study. No patient had been vaccinated against SARS-CoV-2. Table 1 shows the demographic and clinical characteristics of the patients.

**Table 1:** Demographic and clinical characteristics according to COVID-19 symptoms severity (N = 93)

Characteristic		Total	CI 95%	Group 1	CI 95%	Group 1	CI 95%	Group 1	CI 95%	Group 1	CI 95%
		**[n (%)]**		(n = 23)		(n = 24)		(n = 24)		(n = 22)	
				[n (%)]		[n (%)]		[n (%)]		[n (%)]	
Sex											
	Female	27 (29.0)	19.8-38.3	14 (60.9)	40.9-80.8	5 (20.8)	0.0-19.4	2 (8.3)	0.0-19.4	6 (27.3)	8.7-45.9
	Male	66 (71.0)	61.7-80.2	9 (39.1)	19.2-59.1	19 (79.2)	62.9-95.4	22 (91.7)	80.6-100	16 (72.7)	54.1-91.3
Age range (years)											
	18-40	28 (30.1)	28.8-39.4	15 (65.2)	45.8-84.7	6 (25.0)	7.7-42.3	4 (16.7)	7.6-31.6	3 (13.6)	0.0-28.0
	41-60	23 (24.7)	16.6-32.8	7 (30.4)	11.6-49.2	5 (20.8)	0.0-19.4	6 (25.0)	7.7-42.3	5 (22.7)	5.2-40.2
	61-90	42 (45.2)	35.8-54.5	1 (4.3)	0.0-12.7	13 (54.2)	34.2-74.1	14 (58.3)	38.6-78.1	14 (63.6)	43.5-83.7
HCoV status											
	HCoV Seropositivity	69 (74.2)	65.3-83.1	21 (91.3)	79.8-100	16 (66.7)	47.8-85.5	18 (75.0)	57.7-92.3	14 (63.6)	43.5-83.7
	HCoV-NL63	62 (66.7)	57.1-76.2	19 (82.6)	67.1-98.1	16 (66.7)	47.8-85.5	14 (58.3)	38.6-78.1	13 (59.1)	38.6-79.6
	HCoV-HKU1	24 (25.8)	16.9-34.7	5 (21.7)	4.9-38.6	5 (20.8)	0.0-19.4	7 (29.2)	11.0-47.4	7(31.8)	12.4-51.3
	Coinfection frequency	17 (18.3)	10.4-26.1	3 (13.0)	0.0-26.8	5 (20.8)	0.0-19.4	3 (12.5)	0.0-25.7	6 (27.3)	8.7-45.9
HCoV OD (450 nm)											
	HCoV-NL63	0.47	0.02-1.44	0.58	0.19-1.17	0.44	0.02-0.93	0.44	0.14-1.44	0.40	0.14-0.82
	HCoV-HKU1	0.17	0.00-1.42	0.17	0.02-1.42	0.14	0.0-0.37	0.17	0.00-0.37	0.19	0.00-0.48
Comorbidities											
	HIV-1 positive	4 (4.3)	0.02-0.84	0	-	3 (12.5)	0.0-25.7	1 (4.2)	1.8-31.6	0	-
	Hypertension	23 (24.7)	18.8-37.1	2 (8.7)	0.0-20.2	7 (29.2)	11.0-47.4	7 (29.2)	11.0-47.4	7(31.8)	12.4-51.2
	Diabetes mellitus	15 (16.1)	8.7-23.6	0	-	4 (16.7)	7.6-31.6	7 (29.2)	11.0-47.4	4 (18.2)	2.1-34.3
	Tuberculosis	3 (3.2)	0.0-6.8	0	-	1 (4.2)	1.8-31.6	0	-	2 (9.1)	0.0-21.1
	Renal insufficiency	8 (8.6)	2.9-14.3	0	-	0	-	2 (8.3)	0-19.4	6 (27.3)	8.7-45.9
	Obesity	9 (9.7)	3.7-15.7	4 (17.4)	1.9-32.9	1 (4.2)	1.8-31.6	2 (8.3)	0-19.4	2 (9.1)	0.0-21.1
	Pneumonia	7 (7.5)	2.2-12.9	0	-	1 (4.2)	1.8-31.6	2 (8.3)	0-19.4	4 (18.2)	2.1-34.3
	COPD	5 (5.4)	0.8-10.0	0	-	2 (8.3)	0.0-19.4	2 (8.3)	0-19.4	1 (4.5)	0.0-1.25
	More than one comorbidity	17 (18.3)	11.0-25.5	0	-	4 (16.7)	7.6-31.6	6 (25.0)	7.7-42.3	7(31.8)	12.4-51.2
	At least one comorbidity	64 (67.7)	60.1-77.5	6 (26.1)	8.1-44.0	19 (79.2)	62.9-95.4	18 (75.0)	57.7-92.3	21 (95.5)	86.8-100.0

Group 1: asymptomatic patients; Group 2: hospitalized patients; Group 3: patients in the intensive care unit; Group 4: dead patients

ICU: Intensive Care Unit; OD: Optical Density; HIV-1: Human Immunodeficiency Virus; COPD: Chronic Obstructive Pulmonary Disease; CI: Confidence Interval

### Case definition

*Asymptomatic patient (group 1):* Laboratory confirmation of SARS-CoV-2 infection by RT-qPCR without any signs or symptoms commonly associated with the COVID-19 infection.

*Hospitalized patient with moderate signs (group 2):* Hospitalized patient with laboratory confirmation of SARS-CoV-2 infection by RT-qPCR with acute onset of any three or more of the following signs or symptoms: fever, cough, general weakness/fatigue, headache, myalgia, sore throat, coryza, dyspnea, nausea, diarrhea, or anorexia.

*Hospitalized patient in intensive care unit with severe symptoms (group 3):* Patient with laboratory confirmation of SARS-CoV-2 infection by RT-qPCR with severe pneumonia, having acute respiratory distress syndrome, septic shock, respiratory failure, or multi-organ dysfunction or failure.

*Dead patient (group 4):* Patient with laboratory confirmation of SARS-CoV-2 infection using RT-qPCR whose death resulted from a clinically compatible illness with confirmed COVID-19 case unless a clear alternative cause of death that cannot be related to COVID-19 disease *(e.g.,* trauma).

*Patients with negative results for SARS-CoV-2:* Patients with apparent COVID-19 signs or symptoms but with negative RT-qPCR results for SARS-CoV-2.

*Pregnant women:* Female patient with laboratory confirmation of SARS-CoV-2 infection using RT-qPCR and a quantitative blood test confirmation of pregnancy.

### Immunoassays

#### Determination of IgG antibodies against HCoV-NL63

An immunoassay test was performed using the in-house ELISA technique, based on the studies conducted by Lesmes-Rodríguez *et al.* ^23^, Dijkman *et al.* ^27^, and Edridge *et al.* ^28^. Briefly, 3 μg/ml of recombinant nucleocapsid (N) protein (ab270843 Abcam, UK) were used to sensitize the high-ligation ELISA plate (Thermo Fisher, USA). In total, 100 μl of the reconstructed protein were added to each 96 well. The incubation was performed overnight using a humid chamber at 4 °C. The next day, the plate was washed three times with a washing solution (1% PBS + 0.05% Tween-20) and once with 1% PBS. The plate was then blocked with 100 μl of the following solution: 1% PBS + 0.05% Tween-20 + 10% fetal bovine serum (FBS) and incubated at room temperature for one hour. The plate was subsequently washed three times with the washing solution and once with 1% PBS. Each aliquot of serum was diluted (1:500) in 1% PBS + 0.05% Tween-20 + 2,5% FBS.

Subsequently, 50 μl of this solution were applied to each well and incubated at room temperature for one hour. The plate was later washed five times with the washing solution and once with 1% PBS. Then, the plate was incubated at room temperature for one hour with an enzyme solution containing horseradish peroxidase (HRP) conjugated with goat anti-human IgG (Sigma-Aldrich, USA), diluted (1:3,000) in 1% PBS + 0.05% Tween-20 + 2.5% FBS. After that, the plate was washed five times with the washing solution and once with 1% PBS. Then, 50 μl of 3,3',5,5'-tetramethylbenzidine (TMB) (Cell Signaling, USA) were added to each well, and the plate was incubated in the dark for 30 minutes. Next, the reaction was stopped by adding 50 μl of 2N H_2_SO_4_ to each well.

Finally, the optical density was read in the ELx808™ equipment (BioTek Instruments^®^, Inc. Winooski, USA), using a wavelength of 450 nm. Three negative controls were placed containing the mentioned reagents, except the blood sera. The determined cut-off point was 0.348, corresponding to two standard deviations above the mean of the optical density of 12 pre-pandemic sera.

#### Determination of IgG antibodies against HCoV-HKU1

The test was performed using the commercial indirect ELISA kit HCoV-HKU1 with the antibody anti-HCoV-HKU-39849 NP (MyBiosource, San Diego, California, USA). Briefly, 50 μl of each blood serum (undiluted) and control (two negative and one positive) samples were added to the kit plate already sensitized with the HCoV-HKU1 nucleoprotein. Next, 100 μl of an enzyme solution containing horseradish peroxidase (HRP)-conjugated with goat antihuman IgG were added to each well and incubated at 37 °C for one hour. Subsequently, the plate was washed four times with the washing solution, and 50 μl of chromogen A and 50 μl of chromogen B were added to each well. Incubation was performed in the dark for fifteen minutes at 37 °C. Then, 50 μl of the blocking solution was added to each well.

Finally, the optical density was read in the ELx808™ equipment (BioTek Instruments^®^ Inc., Winooski, VT, USA), with a wavelength of 450 nm. The determined cut-off point was 0.213, corresponding to the mean of the optical density of the negative controls plus 0.15. The test was validated with a sensitivity and specificity of 85%.

### Statistical analysis

The seroprevalence (P) of HCoV-NL63 and HCoV-HKU1 in SARS-CoV-2 positive patients was calculated proportionally using the following formula: P = Number of seropositive cases / sample size ^23^. Seroprevalence was expressed as a percentage, with a 95% confidence interval (CI).

Descriptive statistics were used to describe data associated with the different clinical conditions of COVID-19 (asymptomatic patients, hospitalized patients with mild or moderate signs, patients hospitalized in intensive care unit with severe symptoms, and deceased patients), according to the demographic and other clinical characteristics. The study variables and their categories were defined as: Sex (female and male); age range (18-40, 4160, 61-90 years); HCoV status (general HCoV seropositivity, HCoV-NL63 seropositivity, HCoV- HKU1 seropositivity, coinfection frequency); HCoV OD, HIV-1 status (HIV-1 seropositivity); comorbidities frequency (hypertension, diabetes, renal insufficiency, obesity, pneumonia, and chronic obstructive pulmonary disease [COPD]).

Odds ratio (OR) was used as a measure of association to determine whether seropositivity to one (or both) HCoV was related to the severity or absence of clinical signs present in SARS-CoV-2 infections, calculated through a 2 x 2 contingency table (crosstab) analysis, with 95% CI using the SPSS™ software, version 29 (IBM Corp, New York, NY, USA).

Differences between the four groups of patients were assessed using the chi square, Fisher exact, or Mann-Whitney U tests, as appropriate: chi-square or Fishers exact test for categorical variables and Mann-Whitney U test for continuous variables. p values less than 0.05 were considered significant. Graphs were made with the Prism software, version 9.4.1 (GraphPad Software Inc., San Diego, CA, USA).-

### Ethical considerations

The present study was conducted following the standards of the Helsinki Declaration: "Ethical principles for medical research in human beings" 29 and the ethical regulations of the *Ministerio de Salud de Colombia:* "Scientific, technical and administrative standards for health research" established by Resolution 8430 of 1993 30. The present research had the favorable technical concept granted by the Bioethics Committee of the *Universidad de los Llanos,* according to the minute 02 by consensus of April 6, 2021, and the approval concept from the research ethics committee of the *Hospital Departamental de Villavicencio,* according to the ordinance issued on February 8, 2021.

## Results

### Clinical characteristics and seroprevalence of HCoV

The clinical and demographic characteristics of all patients are shown in table 1. Most were men (71.0%) with a mean age of 58.5 years, whereas women represented the 29.0% with a mean age of 46.7 years. The mean age was 55,1 years, with an age range of 18 to 90 years. The overall seroprevalence of IgG antibodies against HCoV was 74.2% (n = 69; 95% CI: 65.3-83.1). The 66.7% corresponded to HCoV-NL63 (n = 62; 95% CI: 57.176.2) and 25.8% to HCoV-HKU1 (n = 24; 95% CI: 16.9-34.7).

Regarding the percentages of coinfection, 17 patients (18.3%) were exposed to the two coronaviruses studied, 52 (55.9%) were exposed to only one coronavirus, and 24 (15.8%) were seronegative for HCoV. The most prevalent comorbidity was hypertension (24.7%), followed by diabetes (16.1%) and obesity (9.7%). It is worth mentioning that 18.3% of the patients exhibited more than one comorbidity, and 67.7% had at least one (table 1). The statistical differences between the four groups of patients (p values) are shown in the supplementary table 1. The comparisons between groups G2 (hospitalized patients), G3 (intensive care unit patients), and G4 (dead patients) did not indicate significant differences.

When groups 2, 3, and 4 were considered together as a single symptomatic group (G2 + G3 + G4) and were compared with group 1 (asymptomatic), we found statistically significant differences in the parameters of sex, age, HCoV seropositivity, optical density of HCoV-NL63 and HCoV-HKU1, diabetes-related comorbidity, and presenting one or more comorbidities (table 1 and table S1).

### Association between antibodies prevalence against coronaviruses HCoV-NL63 and HCoV-HKU1 and the severity of signs of COVID-19

**Figure 1:**
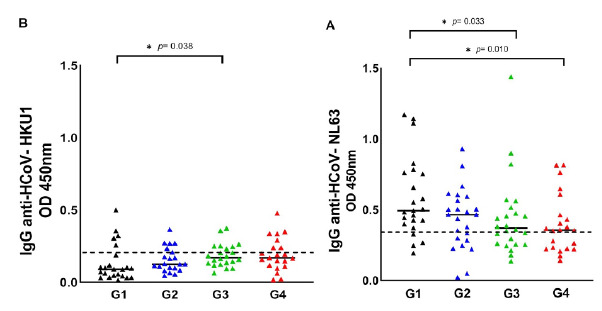
Seroprevalence of HCoV-NL63 and HCoV-HKU1 in the four COVID-19 patient groups. HCoV N-specific IgG antibodies were detected in the participants' serum using indirect ELISA. Results are represented in OD units. (A) IgG anti-HCoV-NL63 (cut-off = 0.348); (B) IgG anti-HCoV-HKU1 (cut-off = 0.213). The cut-off is the dotted horizontal line in the individual figures. Significance was considered as p < 0.05.

G1: asymptomatic patients, G2: hospitalized patients, G3: patients in intensive care units; G4: dead patients

**Figure S1:**
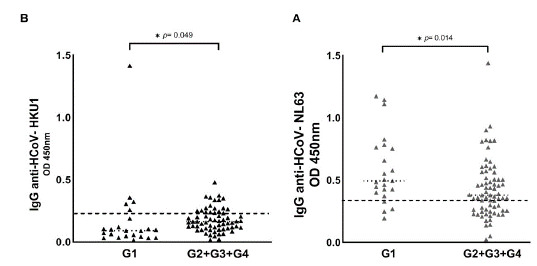
Seroprevalence of HCoV-NL63 and HCoV-HKU1 in the four COVID-19 patient groups. HCoV N-specific IgG antibodies were detected in the participants' serum using indirect ELISA. Results are represented in optical density units. (A) IgG anti-HCoV-NL63 (cut-off = 0.348); (B) IgG anti-HCoV-HKU1 (cut-off = 0.213). The cut-off is the dotted horizontal line in the individual figures. Significance was considered as p < 0.05.

Figure 1 shows the HCoV seropositive patients distributed in each group of COVID-19 patients, namely: G1 (asymptomatic patients), G2 (hospitalized patients), G3 (ICU patients), and G4 (dead patients). Seropositive patients for HCoV are shown in figure S1, comparing the G1 with the symptomatic groups (G2 + G3 + G4). We observed that the optical density of IgG antibodies against the nucleocapsid (N) protein of HCoV-NL63 was statistically higher in asymptomatic patients (table 1, table S1, figure 1A, figure S1A). Regarding HCoV-HKU1, we observed that the optical density of IgG antibodies was statistically higher in patients with severe signs of COVID-19 (table 1, table S1, figure 1B, figure S1B).

**Figure 2:**
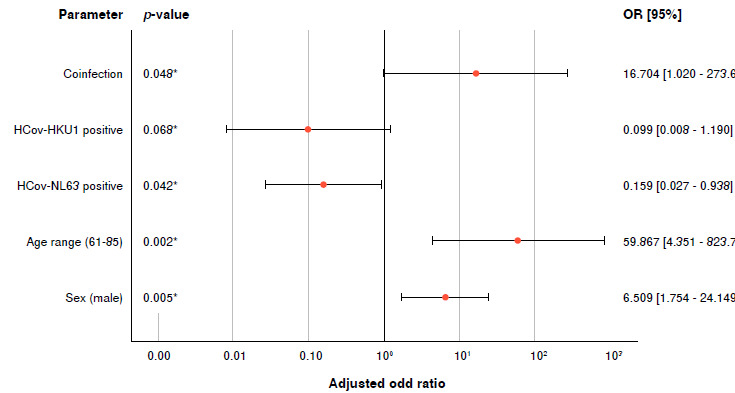
Association between COVID-19 severity and the parameters of coinfection frequency, seropositivity to HCoV-NL63 and HC0V-HKUI, age range (61 -90 years), and male sex. p < 0.05 is considered significant and indicated with an asterisk (*).

We performed a crosstab analysis to determine whether the significant differences between the optical densities of the antibodies against HCoV were related to the increase or decrease in the severity of signs and symptoms of COVID-19 patients (table 2, figure 2). The variables used corresponded to the parameters of interest or with statistically significant differences when comparing the groups of patients.

**Table 2:** Crosstab for the result patients with severe signs of COVID-19 (n = 70, 75.3%)

Characteristic	Patients with severe siqns of COVID-19	OR	95% CI	Adjusted OR	95% CI	P
Sex (male)^3^	57/66 (86.4)	6.821	2.431-19.135*	6.509	1.754-24.149*	0.005*
Age range	41/42 (97.6)					
(61 - 90)^b^		31.103	3.965-243.9*	59.867	4.351-823.729	0.002*
HCoV-NL63 positive^c^	43/62 (69.4)	0.335	0.103-1.092	0.159	0.027-0.938	0.042*
HC0V-HKUI positive^c^	19/24 (79.2)	15.374	0.887-266.5	0.099	0.008-1.190	0.068
Coinfection frequency^d.^	14/17 (82.4)	1.667	0.433-6.413	16.704	1.020-273.670	0.048*

OR: odds ratio; CI: confidence interval

^a^ Male sex is compared to female.

^b^ Age range of 61-90 is compared to 18-40 range.

^c^ HCoV seropositivity is compared to HCoV seronegativity.

^d^ Coinfection frequency is compared to no coinfection.

* Unadjusted and adjusted OR are considered significant when the confidence interval does not contain number 1 (p < 0.05).

As a result, previous exposure to HCoV-NL63 was associated with protection against severe COVID-19 compared to individuals not previously exposed to HCoV-NL63. Individuals exposed to this Coronavirus were 6.3 times more likely not to have severe signs and symptoms of COVID-19 (p = 0.042; adjusted OR = 0.159; 95% CI: 0.027-0.938). The factors associated were: (a) being male: compared to women, men are 6.5 times more likely to exhibit severe COVID-19 disease (p = 0.005; adjusted OR = 6.509; 95% CI: 1.754-24.149); (b) belonging to the age range between 61 and 90 years: in comparison to someone between 18 and 40 years, an individual older than 61 years is 59.9 times more likely to be hospitalized for COVID-19 (p = 0.002; adjusted OR = 59.867; 95% CI: 4.351-823.729); and (c) underlying exposure to the two HCoV: in comparison to individuals with no prior exposure to NL63-HKU1, individuals who have had this coinfection are 16.7 times more likely to have severe COVID-19 disease (p = 0.048; adjusted OR = 16.704; 95% CI: 1.020-273.670).

Individual pre-infection with HC0V-HKUI Coronavirus was not have a statistically significant association with COVID severity (table 2, figure 2). A crosstab analysis was performed to determine the parameters associated with the outcome "dead patients" (n = 22; 23.7%), and no significant associations were found regarding the variables related to HCoV seropositivity. However, advanced age was associated with dying from COVID-19 disease: An individual older than 61 years is 3.8 times more likely to die from COVID-19 disease (p = 0.043; adjusted OR = 3.743; 95% CI: 1.033-8.782]) than one between 18 and 40 years old (table S2).

## Discussion

The present study addressed the seroprevalence of two HCoV and their association with the signs in SARS-CoV-2 infected patients, contributing to the knowledge about the implications of exposure and epidemiological permanence of HCoV in Villavicencio. Several seroprevalence studies of endemic coronaviruses have been conducted worldwide. However, information is missing from Latin America, Europe, and Oceania. To the authors' knowledge, this is the first study addressing HCoV seroprevalence in Colombia and Latin America, finding a prevalence of IgG antibodies against HCoV of 74.2% (95% CI: 65.3-83.1) in COVID-19 patients from Villavicencio (table 1). In general, among endemic coronaviruses, HCoV-NL63 has been one of the most frequent in the adult population (20,23,31,32), whereas HCoV-HKU1 is one of the least frequent (31,33-35). In the present study, HCoV-NL63 was found to be also the most frequent (66.7%; 95% CI: 57.1-76.2), and HCoV-HKU1 was found in a lower proportion (25.8%; 95% CI: 16.9-34.7) (table 1).

The seroprevalence of IgG antibodies against the HCoV N protein varies widely depending on the geographic area and immunodetection methods. In North America, studies are concentrated in the United States and Canada. In the United States, using an in-house ELISA, Severance *et al.* ^31^ and Gorse *et al.* ^33^ found a seroprevalence of anti-HCoV-NL63 of 91.8 - 59.2%, and of anti-HCoV-HKU1 of 98.0 - 91.0%. In Canada, Galipeau *et al.* ^34^ found a seroprevalence of 74.6 and 60.7%, respectively. In Africa, seroprevalences of 90.0% for HCoV-NL63 and 15.0% for HCoV-HKU1 were obtained in Zambia and Tanzania using immunofluorescence assays ^20^. In South Africa, using an *in-house* ELISA, Lesmes-Rodríguez *et al.* ^23^ found seroprevalence of 37.1 and 22.6%, respectively. Seroprevalences of 6.5% and 13.0% were found in Qatar using the PhIP-Seq technique ^32^. Finally, in China, using a competitive ELISA, Gao *et al.* ^35^ determined a seroprevalence of 67.1 and 25.7%, respectively. The results of the present study are within the frequency ranges described by other authors, namely: seroprevalence of 66.7% for HCoV-NL63 (66,7%; 6,5-91,8%) ^32^^,^^31^ and HCoV-HKU1 (25,8%; 13,0-91,0%) ^32^^,^^33^.

HCoV serology indicated that the antibody titers for HCoV-NL63 were significantly higher (p = 0.0329) in the group 1 of asymptomatic patients (median = 0.58; 95% CI: 0.19-1.17) versus the group 3 of patients with severe signs of COVID-19 (median = 0.44; 95% CI: 0.14-1.44) (table 1, table S1, figure S1). Likewise, the crosstab analysis revealed a significant association protective significant from exposure to HCoV-NL63 with severe COVID-19 (p = 0.042; adjusted OR = 0.159; 95% CI: 0.027-0.938) (table 2, figure 2). Other studies have reported contrasting results, *e.g.,* Sagar *et al.* ^36^ found significantly fewer underlying HCoV infections in ICU patients hospitalized due to COVID-19 without distinction between the four HCoV.

Dugas *et al.* (^37)^ found that the lack of IgG antibodies against the N protein of HCoV-OC43 correlated with the risk of critical COVID-19. Gouma *et al.* ^38^^)^ suggested that recent ßHCoV infections (HCoV-HKU1 and HCoV-OC43) potentially limited the duration SARS-CoV-2 infection symptoms, and Lavell *et al.* ^39^ found that previous HCoV-OC43 infections could be associated with protection from SARS-CoV-2 infections. Interestingly, other studies have mentioned that specific exposure to HCoV-NL63 could protect against SARS-CoV-2 infection both in animal models (mice) ^40^ and in hospitalized COVID-19 patients ^23^. Additionally, Galipeau *et al.* ^34^ determined IgG antibodies against HCoV-NL63 and HCoV-OC43 as the most important predictors of neutralization against SARS-CoV-2. Regarding the duration of HCoV antibodies, Edridge *et al.* ^28^ affirmed they were of "short duration" (approximately 12 months), and according to Rees *et al.* ^41^, they remained between 0.9 and 3.8 years. For this reason, in the present study, we hypothesized that the antibodies detected in the performed assays came from relatively recent infections.

However, there are also studies about possible associations (positive or negative) between previous exposures to HCoV and the severity of COVID-19. Anderson *et al.* ^42^ found that HCoV antibodies were enhanced by SARS-CoV-2 infection, though not associated with protection. Likewise, the results obtained by Imai *et al.* ^43^ did not indicate an association between HCoV antibody titers in the early phase of COVID-19 and disease severity. On the other hand, Sayama *et al.* ^44^ performed neutralization assays in samples from a pediatric population and concluded that the reactivity of HCoV antibodies against the S (spike) protein of SARS-CoV-2 did not correlate with neutralization capacity.

Some studies have considered previous HCoV infections as risk factors for severe COVID-19 disease ^23^^,^^45^^,^^46^. Those studies reported that original antigenic sin was the possible cause of the negative association. This immunological phenomenon refers to a low-intensity antibody response against a different antigenic variety of the same virus ^47^, creating an antibody-dependent potentiation, promoting the entry of the virus into the cells and, thereby, intensifying the inflammatory process against secondary infection ^48^. On the other hand, Lin *et al.* ^49^ found that pre-existing humoral immunity to HCoV negatively impacted the protective antibody response against SARS-CoV-2, and Odendahl *et al.* ^50^ stated that humoral immunity in convalescent patients with mild COVID-19 disease was negatively correlated with aHCoV antibody titers. In these latter studies, the phenomenon of immunological imprinting was pointed out as a possible cause of the negative correlation. Ma *et al.* ^51^ defined immunological imprinting as a lifelong bias in immunological memory and protection against subsequent infections. The ability to combat a virus depends not only on the virus subtypes the patient has been exposed to but also on their finding sequence. Whichever subtype is detected by the immune system, an imprint is established. It protects particularly well against strains of the same subtype, though poorly against strains of other subtypes later encountered.

In the present study, prior exposure to coinfection (HCoV-NL63/ HCoV-HKU1) was associated with increased severity of COVID-19 disease (p = 0.048; adjusted OR = 16.704; 95% CI: 1.020-273.670) (table 2, figure 2). In the phenomenon of antibody-dependent enhancement, the production of cross-reactive antibodies with sub-neutralizing capacity exacerbates the inflammatory response and facilitates the entry of the virus into the host cells ^25^ through the interaction with Fc receptors (from crossed antibodies) or the complement system. This process triggers signal transductions that release inflammatory cytokines and generate oxidative burst and antibody-dependent cytotoxicity mediated by leukocytes ^52^. According to this, we hypothesized the probable association of the antibody-dependent enhancement with the severity of clinical signs of COVID-19 disease.

**Figure S2:**
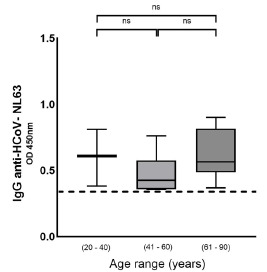
Titers of HCoV-NL63 seropositive patients in the age ranges: 18-40, 41-60, and 61-90 years. Results were considered significant if p < 0.05.

On the other hand, most patients with antibodies against HCoV-NL63 were found in the group of asymptomatic patients (n = 19/62; 30.6%), the majority ranging from 18 to 40 years (n = 15/23; 65.2%). For this reason, we decided to confirm previous exposure to HCoV-NL63 as a protective factor, ruling out age. To that end, HCoV-NL63 titers were stratified by age without finding significant differences (table S3 and figure S2).

Finally, the present study corroborates other widely documented associated factors with severe COVID-19 disease. For example, being male was associated with disease severity (p = 0.005; adjusted OR = 6.509: 95% CI: 1.754 - 24.149) (table 2, figure 2). Vahidy *et al.* ^53^ mentioned that regardless of age, men were more likely to suffer from COVID-19 disease and exhibited more complications since women exhibited more robust immune responses (activation of T cells). Kragholm *et al.* ^54^ found that men exposed to SARS-CoV-2 had greater chances (over 50%) of dying from COVID-19 disease. On the other hand, numerous studies have indicated the association between advanced age and a negative outcome for COVID-19 due to alterations in the structure and function of the immune system ^55^^-^^57^. Likewise, the present study confirmed this association for severe COVID-19 disease in patients over 61 years (p = 0.002; adjusted OR = 59.867; 95% CI: 4.351-823.729) (table 2, figure 2), and also, an association with death (p = 0.043; adjusted OR = 3.012; 95% CI: 1.033-8.782) (table S2).

The experimental design of the present study was performed in August 2020. For this reason, the sample size was calculated on that date, considering the (relatively low) number of COVID-19 patients in Villavicencio. Therefore, the sample size was a limitation of the present study and may affect generalizability. Another limitation is the lack of a cross-reactivity test to confirm that the seroprevalence data was specific for each HCoV. Nevertheless, the ELISA kit with the anti-HCoV-HKU-39849 NP antibody (HCoV-HKU1) (MyBiosource, San Diego, California, USA) was validated with a sensitivity and specificity of 85%. Future studies are required to verify if the reactive HCoV antibodies are neutralizing SARS-CoV-2. In addition, studies using molecular diagnoses (PCR) or direct ELISA diagnostic tests of HCoV viruses in patients with active respiratory symptoms are recommended.

Considering that the onset of pandemics caused by emerging coronaviruses is viable and highly probable due poor control of zoonotic disease transmission, it is important to continue conducting further seroepidemiological studies addressing HCoV. At the same time, it is also imperative to investigate the immunological interactions between emerging and re-emerging endemic coronaviruses to expand epidemiological knowledge about them.

In conclusion, the overall seroprevalence of IgG antibodies against HCoV was 74.2% (95% CI: 65.3-83.1), with 66.7% of HCoV-NL63 (95% CI: 57.1- 76.2) and 25.8% of HCoV-HKU1 (95% CI: 16.9-34.7). To the authors' knowledge, this is the first study addressing the seroprevalence of IgG antibodies against HCoV in Colombia and Latin America. Patients with underlying HCoV-NL63 and HCoV-HKU1 coinfection could be hospitalized with severe signs of COVID-19 disease.

## Archivos suplementarios

**Table S1:** Significant differences (p values) between the four patient groups are shown per characteristic. Fisher's exact test was performed for categorical variables and the Mann-Whitney U test for continuous variables. p values were considered significant if they were less than 0.05.

Characteristic	G1 vs. G2	G1 vs. G3	G1 vs. G4	G2 vs. G3	G2 vs. G4	G3 vs. G4	G1 vs. (G2 + G3 + G4)
Sex	0.0077*	0.0002*	0.0361*	0.4158	0.7343	0.1278	0.0003*
Age range (18-40)	0.0084*	0.0010*	0.0007*	0.7238	0.4638	1.0000	0.0001*
Age range (41-60)	0.5171	0.7516	0.7381	1.0000	1.0000	1.0000	0.5782
Age range (61-90)	0.0003	0.0001	0.0001	1.0000	0.5616	0.7693	0.0001*
HCoV seropositivity	0.0723	0.2448	0.0351*	0.7516	1.0000	0.5252	0.0315*
HCoV-NL63 positive	0.3177	0.1107	0.1075	0.7661	0.7610	1.0000	0.0766
HCoV-HKU1 positive	1.0000	0.7400	0.5136	0.7400	0.5077	1.0000	0.7851
Coinfection frequency	0.7008	1.0000	0.2837	0.7008	0.7343	0.2757	0.5488
HCoV-NL63 OD	0.1870	0.0329*	0.0102*	0.4552	0.2625	0.5526	0.0139*
HCoV-HKU1 OD	0.3777	0.0387*	0.0664	0.1576	0.1402	0.8319	0.0499*
HIV-1 positive	0.2340	1.0000	1.0000	0.6085	0.2348	1.0000	0.5688
Hypertension	0.1365	0.1365	0.0706	1.0000	1.0000	1.0000	0.0512
Diabetes	0.1092	0.0094*	0.0491*	0.4936	1.0000	0.4966	0.0183*
Tuberculosis	1.0000	1.0000	0.2333	1.0000	0.6000	0.2232	0.5720
Renal insufficiency	1.0000	0.4894	0.0092*	0.4894	0.0080*	0.1278	0.1926
Obesity	0.1882	0.4158	0.6652	1.0000	0.6000	1.0000	0.2175
Pneumonia	1.0000	0.4894	0.0092 *	1.0000	0.1783	0.4052	0.1871
COPD	0.4894	0.4894	0.4889	1.0000	1.0000	1.0000	0.3277
More than one comorbidity	0.1092	0.0219*	0.0038*	0.7238	0.3068	0.7462	0.0099*
At least one comorbidity	0.0004*	0.0012*	0.0001*	1.0000	0.1896	0.0984	0.0001*

G1: asymptomatic patients; G2: hospitalized patients; G3, patients in the intensive care unit; G4: dead patients OD: Optical Density; HIV-1: Human Immunodeficiency Virus; COPD: Chronic Obstructive Pulmonary Disease

**Table S2:** Crosstab for the group 4 (G4): dead patients (n = 22; 23.7%)

Characteristic	G4	Unadjusted	95% CI	Adjusted	95% CI	p
		OR		OR		
Sex (male)^a^	16/66 (24.2)	1.120	0.385-3.260	0.773	0.242-2.472	0.664
Age range	14/42 (33.3)	2.688	0.998-7.237	3.012	1.033-8.782*	0.043*
(61-90)^b^						
HCoV-NL63 positive^c^	13/62 (21.0)	0.335	0.649-1.741	0.574	0.186-1.776	0.335
HCoV-HKU1 positive^c^	7/24 (29.2)	1.482	0.519-4.235	0.769	0.139-4.273	0.764
Coinfection frequency^d^	6/17 (35.3)	2.046	0.656-6.379	3.746	0.530-26.500	0.186

OR: odds ratio; CI: confidence interval

^a^ Male sex is compared to female.

^b^ Age range 61-90 is compared to 18-40 range.

^c^ HCoV seropositivity is compared to HCoV seronegativity.

^d^ Coinfection frequency is compared to no coinfection.

* Unadjusted and adjusted OR are considered significant when the confidence interval does not contain the number 1

(p < 0.05).

**Table S3:** Age characteristics of HCoV-NL63 seropositive patients (N = 62) according to COVID-19 severity

Parameter		HCoV-NL63 patients	95% CI G1 [n (%)]	95% CI	(G2 + G3 + G4)	95% CI	G1 vs. (G2 + G3 + G4)
		[n (%)]			[n (%)]		p value
Age range							0.5874 (ns)
	18-40	21 (33.9)	22.1-45.7 12 (63.2)	41.5-84.8	9 (20.9)	8.8-33.1	0.7589 (ns)
	41-60	17 (27.4)	16.3-38.5 6 (31.6)	10.7-52.5	11 (25.6)	12.5-38.6	0.0002*
	61- 0	24 (38.7)	26.6-50.8 1 (5.3)	0.0-15.3	23 (53.5)	38.6-68.4	
	(18-40) vs. (41-60)	p = 1.000 (ns)	p = 0.071 (ns)		p = 0.388 (ns)		
	(18-40) vs. (61-90)	p = 0.203 (ns)	p < 0.001*		p = 0.087 (ns)		
	(41-60) vs. (61-90)	p = 0.282 (ns)	p = 0.006*		p = 0.614 (ns)		

CI: confidence interval; ns: non-significant

G1: asymptomatic patients (n = 19); G2 + G3 + G4: patients with severe COVID-19 signs and symptoms (n = 43)

* p = 0,05

G1 : asymptomatic patients; G2: hospitalized patients; G3, patients in the intensive care unit; G4: dead patients

* p < 0.05

ns: non-significant
